# Assessment of Mental Health in Healthcare Personnel: A Review of DASS, MBI, and Zung Scales

**DOI:** 10.3390/healthcare14132006

**Published:** 2026-07-06

**Authors:** Tanja Lupieri, Martina Hrvačić, Dubravka Švob Štrac, Suzana Uzun, Dunja Degmečić

**Affiliations:** 1 Clinical Hospital “Sveti Duh”, 10000 Zagreb, Croatia; mhrvacic@kbsd.hr; 2 Laboratory for Molecular Neuropsychiatry, Division of Molecular Medicine, Ruđer Bosković Institute, 10000 Zagreb, Croatia; dsvob@irb.hr; 3 Department of Biological Psychiatry and Psychogeriatrics, Reference Center for Alzheimer’s Disease and Psychiatry of Old Age, University Psychiatric Hospital “Vrapče”, 10000 Zagreb, Croatia; suzana.uzun@gmail.com; 4 School of Medicine, University of Zagreb, 10000 Zagreb, Croatia; 5 University Hospital Centre Osijek, 31000 Osijek, Croatia; dunja.degmecic@mefos.hr; 6 Faculty of Medicine Osijek, University of Osijek, 31000 Osijek, Croatia

**Keywords:** healthcare professionals, depression, burnout

## Abstract

**Highlights:**

**What are the main findings?**

- DASS, MBI and Zung SDS are widely used and psychometrically validated instruments for assessing depression, anxiety, stress, and burnout in healthcare professionals.
- DASS provides a multidimensional assessment of psychological distress; MBI is the primary tool for burnout evaluation, while Zung SDS enables rapid screening of depressive symptoms.

**What are the implications of the main findings?**

- DASS, MBI, and Zung SDS instruments may support early detection and monitoring of depression, anxiety, stress, and burnout in healthcare professionals, provided that they are used in an appropriate context and with adequate cultural and linguistic validation as well as appropriate cut-off points.
- Systematic monitoring using validated scales can improve preventive strategies, reduce burnout risk, and enhance the quality of healthcare services.

**Abstract:**

**Background/Objectives:** Healthcare professionals face high occupational stress, emotional burden, and demanding conditions that increase the risk of burnout, depression, and anxiety, with recent post-pandemic evidence indicating a high and rising global prevalence. Early identification of distress is therefore essential. This review compared three widely used instruments—the Depression, Anxiety and Stress Scales (DASS), the Maslach Burnout Inventory (MBI), and the Zung Self-Rating Depression Scale (SDS)—selected because they jointly cover depression, anxiety, stress, and burnout, unlike unidimensional tools (PHQ-9, GAD-7, PSS, CBI) that would need combining for comparable coverage. **Methods:** A narrative integrative review searched PubMed, Scopus, Web of Science, ScienceDirect, and Google Scholar for peer-reviewed articles (English/Croatian) published up to December 2025 (final search: 26 June 2026). Two reviewers independently screened records, resolving disagreements by consensus; quality was appraised narratively. Of 55 records, 35 were included. **Results:** The DASS showed a replicated three-factor structure and strong reliability, with shortened forms (DASS-8, DASS-12) enabling rapid screening at reduced domain coverage. The MBI remained the most widely used burnout measure but was limited by its emotional-exhaustion focus, proprietary licensing, and scoring heterogeneity. The Zung SDS offered a brief depression screen, though dated wording and context-dependent cut-offs favor initial-screen use. Notably, much evidence is derived from non-healthcare populations—the principal limitation. **Conclusions**: The instruments appear complementary rather than interchangeable; their combined use is proposed—as a reasonable suggestion from the literature, not an empirically demonstrated finding—as a multidimensional approach, contingent on context, objective, and appropriate cultural validation.

## 1. Introduction

Healthcare personnel are frequently exposed to substantial psychological burdens due to the demanding nature of their work, long working hours, high emotional involvement with patients, and exposure to life-threatening situations. These conditions can lead to elevated levels of stress, anxiety, depression, and burnout syndrome, which negatively affect both personal well-being and professional performance. Compared to the general population, healthcare professionals are at higher risk of experiencing burnout during their work [1]. Recent systematic reviews and meta-analyses have confirmed the high and rising global prevalence of burnout among healthcare professionals, particularly in the post-pandemic period [2,3]. A global meta-analysis identified key occupational risk factors—including excessive workload, long working hours, and limited organizational support—as significant contributors to burnout syndrome among healthcare workers [2]. Within the public health workforce, pooled estimates indicate that a substantial proportion of workers experience clinically relevant burnout [3], while among intensive-care unit nurses, burnout is closely associated with reduced job satisfaction [4].

Although stress, anxiety, depression, and burnout syndrome frequently overlap in practice and share common symptoms, they represent conceptually distinct phenomena that must be clearly differentiated. Stress is a transient physiological and psychological reaction of the organism to environmental demands (stressors) and is not in itself a pathological state. Anxiety refers to excessive worry, tension, and anticipation of threat, often without a clear external cause, accompanied by somatic symptoms of arousal. Depression is a clinical syndrome characterized by low mood, loss of interest and pleasure, and cognitive and somatic changes persisting over an extended period. Burnout syndrome, in contrast, is a specifically work-related phenomenon arising from chronic exposure to occupational stress, manifested through three dimensions: emotional exhaustion, depersonalization, and reduced personal accomplishment. Distinguishing between these constructs is essential, as they require different approaches to assessment, prevention, and intervention, and their overlap may hinder the timely and accurate identification of psychological difficulties among healthcare workers.

Numerous validated instruments are currently used in mental health assessment, including the Patient Health Questionnaire (PHQ-9) for depression, the Generalized Anxiety Disorder scale (GAD-7) for anxiety, the Perceived Stress Scale (PSS) for perceived stress, and the Copenhagen Burnout Inventory (CBI) for burnout. Although these tools are reliable and widely applied, they typically assess only a single dimension of psychological burden, which requires the simultaneous administration of several separate questionnaires for comprehensive monitoring. For the purposes of this review, DASS, MBI, and Zung SDS were selected for several reasons. DASS was chosen because it simultaneously measures three closely related yet conceptually distinct constructs—depression, anxiety, and stress—within a single instrument, thereby surpassing unidimensional tools such as PHQ-9, GAD-7, or PSS, which would need to be administered together to achieve the same assessment scope. Furthermore, the availability of shortened versions (DASS-21, DASS-12, and DASS-8) enables rapid screening while preserving acceptable psychometric properties. MBI was included because it remains the gold standard and the most widely used instrument for burnout assessment, with an internationally confirmed three-factor structure; although alternatives such as the CBI exist, MBI allows comparability with the largest body of existing research in healthcare populations. Zung SDS was selected as a simple, brief, and self-rating instrument that enables rapid screening of depressive symptoms and, owing to its long-standing use and validation across diverse populations, complements the previous two tools well [5,6,7,8].

In this way, the present review addresses a specific gap in the existing literature: while numerous studies examine individual instruments separately, an integrated comparison of tools that together cover the full spectrum of relevant psychological outcomes in healthcare workers—from depression, anxiety, and stress to burnout—is lacking. Through the combined analysis of DASS, MBI, and Zung SDS, this work aims to offer a multidimensional framework for the early detection and systematic monitoring of healthcare personnel’s mental health. The clinical significance of these phenomena extends beyond occupational well-being; recent evidence has demonstrated an association between burnout and suicidal behaviors among health professionals, with self-esteem acting as a moderating factor [9]. These findings underscore the need for valid and reliable instruments capable of early detection of psychological distress in this population.

The Maslach Burnout Inventory (MBI) is one of the most widely used instruments for assessing burnout among healthcare professionals. This multidimensional tool evaluates three core components of burnout: emotional exhaustion, depersonalization, and reduced personal accomplishment, providing important insights into the relationship between working conditions and healthcare workers’ mental health [10]. Despite its extensive international application, variations in MBI versions, scoring systems, and interpretation criteria represent important methodological challenges that limit comparisons between studies and healthcare systems. Greater standardization of burnout assessment methods is therefore needed to improve understanding of risk factors and support the development of effective preventive strategies [10]. Preventing burnout among healthcare professionals requires a comprehensive approach that combines individual coping strategies with organizational interventions. Improving working conditions, optimizing workload management, strengthening institutional support, and promoting healthcare workers’ psychological well-being are essential steps toward improving healthcare quality and sustainability [10].

In summary, understanding the prevalence and interrelationships of stress, depression, anxiety, and burnout in healthcare settings is essential for safeguarding the mental well-being of healthcare professionals and maintaining the quality of patient care. Accurate assessment of these psychological phenomena enables early detection and timely intervention. Standardized instruments, such as the Depression, Anxiety and Stress Scales (DASS), Maslach Burnout Inventory (MBI), and Zung Self-Rating Depression Scale (SDS), have been widely used in research and clinical practice to quantify mental health outcomes among healthcare workers. In this review, we provide an overview of these tools, highlighting their applicability in identifying individuals at risk and supporting the development of preventive strategies, psychological support, and organizational improvements. Burnout among healthcare workers remains a major challenge for healthcare systems worldwide despite decades of research. Evidence suggests that burnout adversely affects healthcare workers’ psychological and physical well-being, contributes to reduced job satisfaction, increases staff turnover, and may negatively influence the quality and safety of patient care. Recent evidence indicates that burnout is a multifactorial phenomenon resulting from the interaction between organizational and individual determinants. Organizational factors most frequently associated with burnout include excessive workload, long working hours, inadequate organizational support, poor workplace communication, staffing shortages, and work–life imbalance. In addition, individual characteristics such as younger age, lower stress resilience, moral distress, and personality traits, particularly neuroticism, have been identified as important contributors to burnout risk.

## 2. Materials and Methods

### 2.1. Review Design and Methodological Framework

A narrative integrative literature review was conducted to summarize and compare the application of the Depression Anxiety Stress Scales (DASS), the Maslach Burnout Inventory (MBI), and the Zung Self-Rating Depression Scale (SDS) in the assessment of healthcare workers’ mental health. The narrative integrative approach was selected because it enables the synthesis of heterogeneous sources—including validation studies, psychometric analyses, and applied clinical research of various designs—that cannot be meaningfully combined within the more rigorous framework of a systematic review or meta-analysis. As the objective of this study was the conceptual and psychometric comparison of the three instruments rather than the quantitative aggregation of effect sizes, the narrative approach was considered methodologically appropriate. Studies conducted in non-healthcare populations (students, adolescents, older adults, oncology patients) were included solely to document the psychometric properties and cross-cultural validity of the instruments, and not as direct evidence regarding healthcare workers. Such findings substantiate the structural stability and reliability of the scales, which are prerequisites for their application in healthcare settings.

### 2.2. Data Sources and Search Period

A structured literature search was conducted in the following electronic databases: PubMed, Scopus, Web of Science, ScienceDirect, and Google Scholar. The search included studies published up to 31 December 2025. The final search of all databases was performed on 26 June 2026. The following search filters were applied: language (English and Croatian), source type (peer-reviewed scientific articles), and full-text availability. The time filter was limited to studies published up to 2025 to ensure inclusion of recent literature published during and after the COVID-19 pandemic. In addition to the electronic database search, the reference lists of relevant articles were manually screened to identify additional sources not retrieved through electronic searching. Access to certain full-text articles that were not freely available online was obtained through institutional subscriptions of hospital libraries, including the Library of Sveti Duh University Hospital. In this context, hospital libraries were not used as independent sources for literature identification but solely as a means of accessing the full texts of articles that had already been identified through the aforementioned electronic databases.

### 2.3. Search Strategy

The search strategy was based on combinations of keywords connected by Boolean operators (AND and OR). The following search terms were used: (“DASS” OR “Depression Anxiety Stress Scales”) AND (“mental health” OR “depression” OR “anxiety” OR “stress”); (“Maslach Burnout Inventory” OR “MBI”) AND (“burnout” OR “healthcare workers”); (“Zung Self-Rating Depression Scale” OR “Zung SDS”) AND (“depression” OR “screening”). These terms were combined with population-related terms (“healthcare workers” OR “healthcare professionals” OR “nurses” OR “physicians” OR “medical staff”). Where available, database-specific indexing terms (e.g., MeSH terms in PubMed) and truncation symbols (e.g., “burnout*”) were used. The search strategy was adapted to the syntax requirements of each individual database. The complete, database-specific search strings, including all Boolean combinations, indexing terms, and truncation symbols applied to each database, are provided as Supplementary Material (Table S1).

### 2.4. Inclusion and Exclusion Criteria

The inclusion criteria were: (1) peer-reviewed original research articles and review articles; (2) studies published in English or Croatian; (3) studies investigating the psychometric properties, validation, or clinical/applied use of the DASS, MBI, or Zung SDS; (4) studies conducted among healthcare workers or related populations (including the general population, students, older adults, and patients with chronic diseases when relevant to the psychometric validation of the instruments); and (5) studies reporting outcomes related to measures of depression, anxiety, stress, or burnout. Various study designs were considered, including cross-sectional studies, validation studies, cohort studies, and previous reviews. The exclusion criteria were: (1) studies not relevant to the assessment of mental health in occupational or healthcare settings; (2) editorials, letters to the editor, conference abstracts, and non-peer-reviewed sources; (3) studies without available full text; and (4) studies published in languages other than English or Croatian.

### 2.5. Study Selection Process

**Figure 1:**
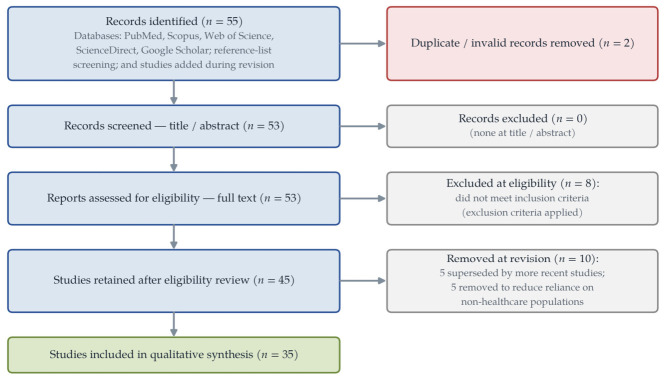
PRISMA flow diagram of the study selection process. Of 55 records (48 identified through database searching and reference-list screening, plus 7 healthcare-worker-specific studies added during revision in response to peer review), 2 duplicate/invalid records were removed, and 8 were excluded at the full-text eligibility stage. Of the studies retained, 10 were removed at revision (5 superseded by more recent studies; 5 removed to reduce reliance on non-healthcare populations), yielding 35 studies included in the qualitative synthesis.

Records identified through the search process were compiled. Two reviewers independently screened titles and abstracts according to the predefined inclusion and exclusion criteria. Potentially relevant articles were subsequently assessed in full text to determine eligibility. Disagreements were resolved through discussion and consensus, and when necessary, with the involvement of a third reviewer. Following this process, a total of 35 studies were included in the qualitative synthesis. The study selection process is illustrated in Figure 1.

### 2.6. Quality Assessment and Data Synthesis

Applying these criteria, the appraised studies varied in methodological strength. Validation studies of the DASS-21 and MBI generally featured adequate-to-large samples, confirmatory factor-analytic designs, and explicit reliability and validity reporting, and were judged of moderate-to-high quality, whereas several studies were limited by convenience sampling, single-site recruitment, or reliance on non-healthcare populations, and were appraised as lower in direct relevance to healthcare workers. Studies employing the Zung SDS were typically smaller and applied the scale as a screening tool rather than validating it, which lowered their methodological weight for the present synthesis. Findings supported by multiple, methodologically stronger studies were given greater interpretive emphasis, while those from weaker or contextually distant studies were treated as indicative rather than confirmatory. Although narrative reviews do not require a formal quantitative assessment of risk of bias comparable to that used in systematic reviews, the methodological quality and relevance of the included studies were critically evaluated throughout the selection and synthesis process. Particular attention was paid to the psychometric rigor of the studies, including sample size and representativeness, reporting of reliability and validity measures, and the appropriateness of statistical analyses, as well as to the consistency of findings across studies. The collected data were synthesized qualitatively by grouping findings according to the instrument studied (DASS, MBI, and Zung SDS) and according to thematic categories such as psychometric properties, cross-cultural validation, and application in specific populations. The methodological quality of the principal included studies was appraised based on study design, sample size, reported reliability, and validity evidence. A structured quality assessment of the principal psychometric and validation studies, organized by instrument, is provided as Supplementary Material (Table S2).

### 2.7. Ethical Considerations

Ethical approval was not required because the study was based exclusively on previously published literature and did not involve the collection of primary data or the participation of human subjects.

## 3. Results

### 3.1. Depression, Anxiety and Stress Scales (DASS)

The Depression, Anxiety and Stress Scales (DASS) were introduced by Lovibond and Lovibond (1995) [11]. Initially developed to assess depression and anxiety, the scales were later expanded to include stress. The original version of the DASS consisted of 42 items, distributed across three subscales of 14 items each, with responses rated on a four-point Likert scale. To facilitate broader application, a shortened version—DASS-21—was subsequently developed while retaining the core structure of the original instrument [12]. Today, several versions of the DASS are available (DASS-42, DASS-21, DASS-12, DASS-8), all demonstrating high reliability and validity. Among these, DASS-21 is the most frequently used version across diverse populations, including healthcare professionals.

#### 3.1.1. Psychometric Properties and Comparison of DASS Versions

A study conducted in Italy compared the psychometric properties of the abbreviated DASS versions (DASS-21, DASS-12, DASS-10, and DASS-8) in the Italian general population. All DASS versions demonstrated high reliability and a negative association with resilience (BRCS), particularly for depression. Among the abbreviated versions, DASS-8 showed the best model fit and the highest explained variance, although it had limited domain coverage. DASS-12 was identified as the best compromise between brevity and structural clarity. DASS-8 has been recommended for rapid screening in primary healthcare and digital platforms, whereas DASS-12 is suggested when symptom differentiation is particularly important [12]. Numerous other studies have also compared these scales. For example, the psychometric properties of DASS-21 were assessed among primary school teachers in Spain and China. Results indicated that DASS-21 is a reliable tool in both countries, although cultural differences affected the factor structure: the one-factor model was more appropriate for Chinese teachers, while the three-factor model better suited Spanish teachers [13]. Comparison of the DASSs is presented in Table 1.

#### 3.1.2. DASS in Clinical Populations

A study comparing DASS-21 psychometric properties between an oncology population (*n* = 376) and a non-cancer control group (*n* = 207) reported that DASS-21 demonstrated a stable factor structure in both groups, with satisfactory internal reliability. Validity was confirmed through expected associations with measures of suicidality, quality of life, and self-rated health, suggesting that DASS-21 is a reliable and valid instrument for assessing psychological distress in cancer patients [14]. Another study in Saudi Arabia assessed the validity and reliability of DASS-21 and its abbreviated versions among the general population and psychiatric patients during the COVID-19 pandemic. Results indicated that DASS-8 was the most stable and reliable version, showing favorable psychometric properties and equal ability to distinguish between clinical and non-clinical participants as the original scale. The authors recommend DASS-8 as a rapid and efficient measure for assessing depression, anxiety, and stress in practice and research [15].

#### 3.1.3. Cross-Cultural Validation of DASS

A study examining the factor structure and reliability of DASS-42, DASS-21, and DASS-12 among adults in Poland confirmed a good three-factor structure and reliability for all versions, along with high internal consistency and adaptability within the Polish context. DASS-21 and DASS-12 demonstrated good validity and can be used as shorter alternatives to DASS-42 for faster assessment without significant loss of information [16].

#### 3.1.4. DASS in Specific Populations and Contexts

Among higher education students in Portugal during the COVID-19 pandemic, DASS-21 also showed high internal consistency and good validity for measuring depression, anxiety, and stress, with symptoms clearly differentiated. These data confirm that DASS-21 is a reliable instrument for monitoring youth mental health in crisis conditions [17]. When psychometric properties of DASS-21 were investigated among university students using item response theory and classical test theory measures, results showed good internal consistency, high factorial validity, and strong discrimination across symptom severity levels. Given significant symptoms of depression, anxiety, and stress among students, DASS-21 is a reliable tool for monitoring mental health under academic pressures [18]. Authors using a bifactor exploratory structural equation modeling (ESEM) approach suggested that DASS-21 has good validity, internal consistency, and a robust factor structure comprising a general factor (covering depression, anxiety, and stress) [19].

Importantly, the DASS-21 has also been validated directly in healthcare workers: in a sample of 1135 hospital nurses, confirmatory factor analysis supported the three-factor structure, with high internal consistency (Cronbach’s α = 0.93 for depression, 0.91 for stress, and 0.79 for anxiety) and acceptable test–retest reliability (ICC 0.75–0.86), confirming it as a valid and reliable instrument for assessing depression, anxiety, and stress among nurses [20]. Applied studies document a substantial symptom burden: among 364 healthcare workers in a Philippine tertiary hospital, depression, anxiety, and stress reached 49.2%, 61.5%, and 30.2% [21], while among 380 Greek hospital nurses one year after the pandemic, 35%, 33.3%, and 33.9% screened positive for depressive, anxiety, and stress symptoms, with the DASS-21 administered alongside the Copenhagen Burnout Inventory [22]. Dedicated validation across other healthcare roles (e.g., physicians, midwives) nevertheless remains limited. Dedicated validation across other healthcare roles (e.g., physicians, midwives) nevertheless remains limited.

### 3.2. Maslach Burnout Inventory (MBI)

Maslach Burnout Inventory (MBI) is the most commonly used instrument for assessing burnout syndrome, encompassing three dimensions: emotional exhaustion, depersonalization, and reduced personal accomplishment (Maslach et al., 1981) [23]. Among healthcare professionals, burnout is associated with chronic stress, overwork, and emotional fatigue [24]. Several versions of the MBI exist—the MBI-HSS (and its adaptation for medical personnel, the MBI-HSS-MP) and the MBI-GS for general occupations—which must be considered, as healthcare workers do not constitute a homogeneous group. Beyond its focus on emotional exhaustion, the MBI has been criticized for its proprietary (licensing) model, the absence of universally accepted cut-off thresholds, and insufficiently established cross-cultural measurement invariance. Comparison with alternative instruments, such as the Copenhagen Burnout Inventory (CBI) and the Oldenburg Burnout Inventory (OLBI), is therefore recommended.

#### 3.2.1. Application of MBI in Healthcare Settings

A study conducted in Brazil across multiple universities and institutions investigated the use of MBI among healthcare workers in public health services. Results indicated that healthcare workers in public health systems are particularly susceptible to burnout due to excessive workloads, emotional stress, and lack of resources. The study highlighted the importance of MBI for early detection of burnout symptoms, as well as proactive preventive and management strategies, including organizational changes, psychological support, and training to improve healthcare workers’ mental health [10].

#### 3.2.2. Psychometric Validation and Cross-Cultural Use of MBI

Analysis of the psychometric properties of MBI for medical personnel (MBI-HSS-MP), conducted in Taiwan, Iran, the UK, and Sweden, confirmed the three-factor structure of MBI, covering emotional exhaustion, depersonalization, and reduced personal accomplishment as key dimensions of burnout. Recent evidence from Peru also supports the reliability and validity of the MBI among healthcare professionals, confirming its applicability for assessing burnout through the dimensions of emotional exhaustion, depersonalization, and reduced personal accomplishment [24]. MBI-HSS-MP proved to be a reliable and universal tool across genders and professional roles, especially for early burnout diagnosis, which can improve working conditions and reduce occupational stress among healthcare professionals [24].

#### 3.2.3. Critical Perspectives and Limitations of MBI

Researchers from the Norwegian University of Science and Technology and the City University of New York critically analyzed MBI, arguing that it is not fully adequate for understanding burnout syndrome. MBI predominantly focuses on emotional exhaustion, neglecting other important burnout aspects such as cognitive and physical consequences. Furthermore, it does not account for broader contextual factors, including organizational and individual characteristics. Therefore, the development of a more comprehensive model encompassing a wider range of burnout dimensions has been recommended [25].

#### 3.2.4. Comparison of MBI with Alternative Burnout Assessment Approaches

A study conducted at the University of California, San Francisco, compared MBI with a simple, self-defined single-item burnout measure among physicians and healthcare staff. Significant differences were observed: MBI often underestimates burnout prevalence, while the simpler, self-defined instrument reports higher rates of burnout. The study suggests that both approaches can be used, but context and specific assessment goals must be considered when choosing a burnout measurement tool [26].

### 3.3. Zung Self-Rating Depression Scale (SDS)

Zung Self-Rating Depression Scale (SDS), developed in 1965, is a standardized instrument for assessing the severity of depression. It consists of 20 items covering emotional and somatic symptoms, with an optimal cut-off score of 40, allowing accurate differentiation between depressed and non-depressed individuals. The self-rating format allows individuals to assess their own depressive symptoms, including sadness, insomnia, and loss of energy. This two-dimensional model is practical, valid, consistent, and reliable for rapid assessment in clinical and research settings, validated across diverse populations including older adults and patients with chronic illnesses [27]. To synthesize the preceding analysis, Table 2 provides a structured side-by-side comparison of the three instruments, summarizing their core characteristics, psychometric properties, respective strengths and limitations, and overall suitability for assessing mental health in healthcare personnel.

#### 3.3.1. Validation and Application of Zung SDS in Various Populations

Validation studies among older adults in Finland and Estonia demonstrated good validity and internal consistency, suggesting Zung SDS as a simple and effective tool for routine primary healthcare use, aiding early detection and care for the elderly [28]. The Zung SDS has also been applied directly in healthcare settings: among 645 medical staff it yielded a one-year depression prevalence of 42.7% [29]; among 800 hospital staff during a local COVID-19 outbreak depression was identified in 35.1%, higher in nurses than physicians [30]; and in 267 staff of a designated COVID-19 hospital depression reached 63.67% [31], with a longitudinal study of medical staff further tracking anxiety and depression over time [32]. Across these studies, however, the Zung SDS was used as a screening tool rather than psychometrically validated in healthcare workers, so dedicated validation in this population remains an open gap. Studies determining the optimal cut-off score confirmed that a value of 40 or higher effectively distinguishes depressed from non-depressed individuals, with discussions of sensitivity and specificity at different thresholds supporting its reliability for depression diagnosis and potential cut-off adjustment based on clinical context [33]. Cheng et al. investigated anxiety and depression prevalence among Chinese patients with rheumatoid arthritis, comparing HADS and Zung SAS/SDS. High prevalence was observed, particularly among women, younger patients, and those with longer disease duration. Good consistency was found between HADS and Zung SDS, indicating both as reliable tools for mental health assessment in comprehensive patient care [34]. These studies underscore the versatility and validity of Zung SDS in older adults, chronic illness patients, and international cohorts, providing guidance on cut-off scores and comparison with alternative instruments for clinical and research contexts.

#### 3.3.2. Contemporary Evaluation and Limitations of Zung Scales

Dunstan et al. demonstrated that widely used Zung SAS and SDS are effective for mental health screening, but their limitations in contemporary assessment contexts necessitate updates and refinements. Zung scales are useful as an initial step in identifying mental disorders; however, combining them with more comprehensive psychological assessments is recommended for greater diagnostic precision [35].

## 4. Discussion

**Figure 2:**
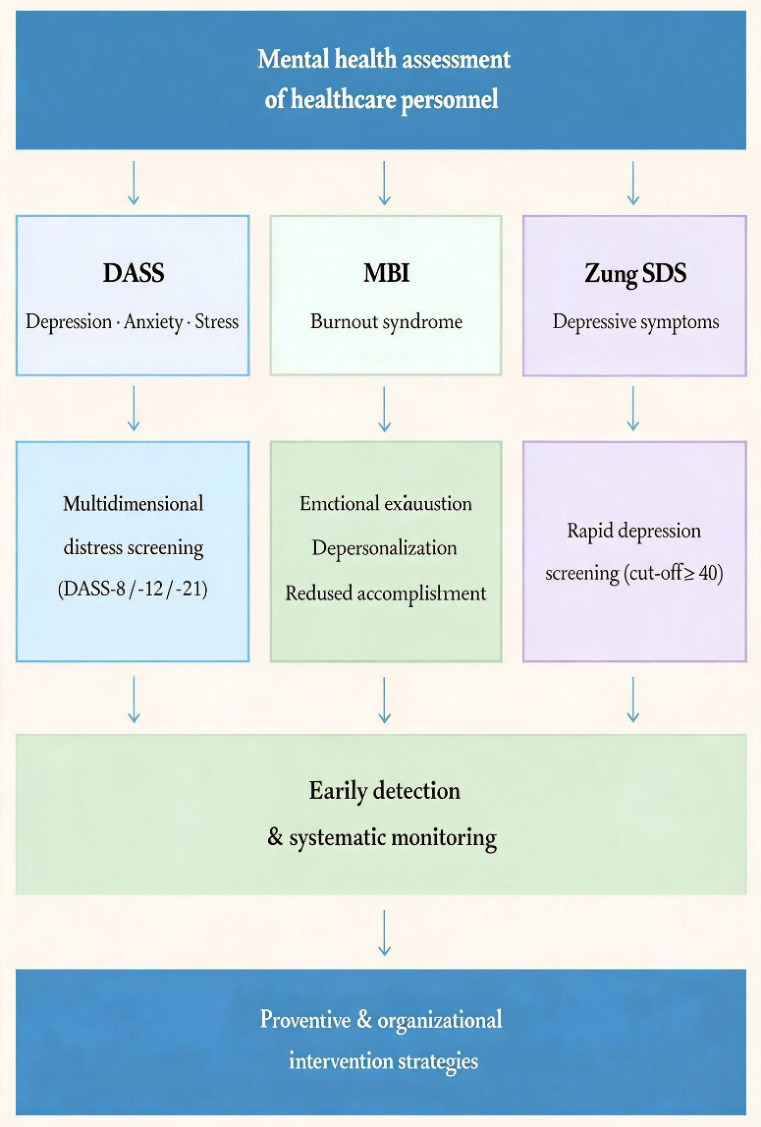
Complementary application of DASS, MBI, and Zung SDS instruments may support early detection and monitoring of mental health among healthcare personnel.

Beyond their measurement properties, the constructs assessed by these instruments carry tangible consequences for health systems. The complementary use of DASS, MBI, and Zung SDS instruments may support early detection and monitoring of mental health among healthcare personnel if they are used in an appropriate context and with adequate cultural and linguistic validation, as well as appropriate cut-off points (Figure 2).

Burnout and untreated psychological distress among healthcare workers have been linked to increased medical errors, reduced quality and safety of care, lower patient satisfaction, absenteeism, and elevated staff turnover, all of which strain already overburdened systems. Anxiety and depression further impair clinical decision-making and empathic engagement, directly affecting patient outcomes. Viewed in this light, the routine, validated assessment of distress is not merely a research exercise but a patient-safety and workforce-sustainability priority, justifying the integration of such instruments into organizational monitoring frameworks.

DASS has proven reliable in assessing depression, anxiety, and stress, not only in healthcare professionals but across various populations and cultural contexts, including students, older adults, and patients with chronic illnesses. Shortened versions such as DASS-8 and DASS-12 are particularly suitable for rapid mental health screening in primary healthcare and digital platforms, while DASS-21 provides a comprehensive evaluation when differentiation among symptoms is important. Although the DASS demonstrates robust reliability and a replicable factor structure, several limitations temper its application in healthcare settings. The frequent dominance of a general distress factor raises questions about the discriminant interpretation of its separate depression, anxiety, and stress scores [22]. Its somatic and stress-related items may also capture fatigue or sleep disruption attributable to shift work rather than psychopathology, inflating scores among healthcare workers. Compared with the PHQ-9 and GAD-7—brief, freely available tools with established clinical cut-offs and diagnostic alignment—the DASS offers broader symptom coverage but less direct diagnostic correspondence, while compared with the PSS it provides multidimensional rather than stress-specific assessment [5,6,7]. These trade-offs should guide instrument selection according to the assessment objective. Reassuringly, recent evidence has begun to offset the earlier reliance on non-healthcare samples: the DASS-21 has been validated directly in hospital nurses, with a confirmed three-factor structure and strong reliability [20], and applied studies document a high symptom burden among healthcare workers in tertiary hospitals [19,21].

Although MBI is the most widely used instrument in assessing burnout and provides valuable insights, some studies highlight its limitations, particularly its focus on emotional exhaustion and lack of consideration for cognitive and physical dimensions of burnout. Therefore, alternative or complementary measures may be necessary for a more holistic assessment of burnout syndrome.

The Zung SDS benefits from brevity, low respondent burden, and an extensive validation history, yet its limitations warrant critical consideration. Its wording and normative thresholds, developed decades ago, may not reflect contemporary symptom expression, and the frequently cited cut-off of 40 is not universally transferable across cultures, languages, or occupational groups [33]. Relative to contemporary depression screens—the PHQ-9, HADS-D, BDI-II, or CES-D—the Zung SDS shows weaker alignment with current diagnostic criteria and fewer recent occupational validation studies [5,33]. Its continued use is therefore best justified as a rapid initial screen, with positive results confirmed by more contemporary, clinically anchored instruments.

On the other hand, the combined application of DASS, MBI, and Zung scales enables a multidimensional assessment of mental health among healthcare personnel. These tools collectively provide a comprehensive framework for early detection of psychological distress, depression, anxiety, and burnout among healthcare professionals, forming the basis for the development of organizational preventive and intervention strategies. Since psychological burden is not restricted by gender, age, specific professional role, or length of service, mental health support should be implemented systematically across all healthcare settings. It must be emphasized that the DASS, MBI, and Zung SDS are screening and symptom-severity measures rather than diagnostic instruments; elevated scores indicate the need for further assessment but do not, in themselves, establish a clinical diagnosis. Moreover, the measured constructs partially overlap—stress on the DASS, emotional exhaustion on the MBI, and depressive symptoms may share common variance. The combined use of the three instruments should therefore be interpreted as complementary, with awareness of potential redundancy.

Our observations are broadly consistent with recent systematic reviews reporting elevated post-pandemic prevalence of burnout [1,2,3,4], depression, and anxiety among healthcare workers, and with prior comparative analyses noting the absence of a single instrument capable of capturing the full distress spectrum [8,9,10]. However, whereas earlier reviews have tended to evaluate burnout and mood instruments in isolation, the present synthesis underscores their complementarity and the practical implications of construct overlap when they are deployed together. This integrative perspective extends, rather than merely reiterates, the existing literature.

## 5. Conclusions

Based on the reviewed literature, it is suggested that the DASS may serve as a brief multidimensional screen for distress, the MBI as a burnout-specific instrument, and the Zung SDS as a rapid screen for depressive symptoms. Their combined application represents a reasonable proposal derived from the literature rather than a conclusion empirically demonstrated by the present work. The utility of each scale depends on the context, the assessment objective, and the availability of appropriate linguistic and cultural validation and cut-off values.

## 6. Limitations

This review has several limitations. First, the narrative design does not include a formal risk-of-bias assessment, leaving the conclusions susceptible to selection bias. Second, the search was restricted to studies published in English and Croatian, introducing potential language bias. Third, a proportion of the included studies were conducted in non-healthcare populations, which limits the direct transferability of the findings to healthcare workers. Finally, the absence of a quantitative synthesis precludes the statistical pooling of results.

## Figures and Tables

**Table 1:** Comparison of DASS versions.

Version	Items (Per Subscale)	Factor Structure/Reliability	Recommended Use
**DASS-42**	42 (14)	Original instrument; robust three-factor structure; high internal consistency	Full assessment and research baseline; longest administration
**DASS-21**	21 (7)	Most widely used version; three-factor structure replicated in systematic review/meta-analysis; subscale reliability typically reported as Cronbach’s α ≈ 0.80–0.94	Standard choice across populations, including healthcare workers, when symptom differentiation matters
**DASS-12**	12 (4)	Retains a good three-factor structure (Polish adults), described as the best compromise between brevity and structural clarity	When symptom differentiation is needed but administration time is limited
**DASS-8**	8	Best model fit and highest explained variance among the short forms, but limited domain coverage; most stable short form in a Saudi general + psychiatric sample	Rapid screening in primary healthcare and digital platforms

**Table 2:** Structured comparison of DASS, MBI, and Zung SDS across key characteristics, psychometric properties, strengths, limitations, and suitability for healthcare personnel.

Attribute	DASS (Focus: DASS-21)	MBI (MBI-HSS/ MBI-HSS-MP)	Zung SDS
**Construct(s)** **measured**	Depression, anxiety, and stress (three negative emotional states)	Burnout	Depression severity
**Developer (year)**	Lovibond & Lovibond (1995) [11]; DASS-21 is the short form of the DASS-42	Maslach & Jackson (1981) [23]; MBI-HSS-MP adapted for medical personnel	Zung (1965) [27]
**Number of items**	21 (7 per subscale); full DASS-42 = 42	22 (MBI-HSS/MBI-HSS-MP)	20
**Domains/** **subscales**	3 subscales: Depression, Anxiety, Stress	3 dimensions: emotional exhaustion, depersonalization, (reduced) personal accomplishment	Affective, cognitive, and somatic symptoms (2–4 factor solutions reported)
**Response format**	4-point Likert (0–3); applies to the past week	7-point frequency scale (0 = never… 6 = every day)	4-point frequency scale (1–4); several items reverse-scored
**Scoring**	Sum 7 items per subscale; multiply by 2 for DASS-42 equivalence	Three separate subscale scores; no single total burnout score	Raw sum (20–80) → SDS Index = raw × 1.25
**Score range**	0–21 raw/0–42 per subscale (doubled)	Subscale-dependent (MBI-HSS: EE 0–54, DP 0–30, PA 0–48)	Raw 20–80/Index 25–100
**Cut-off/** **interpretation**	Severity bands per subscale (e.g., Depression: normal 0–9, mild 10–13, moderate 14–20, severe 21–27, extremely severe ≥28, doubled scores)	Subscale-specific high/moderate/low bands; thresholds vary by version and manual	Index < 50 normal, 50–59 mild, 60–69 moderate–marked, ≥70 severe
**Administration time**	≈5–10 min	≈10–15 min	≈5–10 min
**Licensing/cost**	Free; public domain for research and clinical use (commercial use may require permission from the Psychology Foundation of Australia)	Proprietary (© Maslach & Jackson); licensed/purchased per administration via Mind Garden—not free	Freely reproduced; no central commercial licensor (Zung, 1965 [27])
**Populations** **validated in**	General population, students, adolescents, older adults, oncology and chronic-illness patients, and healthcare workers; broad cross-cultural validation	Healthcare professionals internationally (e.g., Taiwan, Iran, UK, Sweden, Peru, Brazil); across genders and roles	Older adults, chronic-illness patients (e.g., RA, NSCLC), general and clinical populations; international cohorts
**Key strengths**	Three priority constructs in one brief tool; replicated three-factor structure; several validated short forms; free	Gold standard for burnout; internationally confirmed three-factor structure; comparability with the largest body of burnout research	Very short, simple, low reading level; rapid screening; long validation history
**Main limitations**	Subscales are correlated (especially depression–stress); some samples favor a one-factor solution; not diagnostic	Predominantly emotional-exhaustion focused; omits cognitive/physical and contextual dimensions; version/scoring heterogeneity limits cross-study comparison; can underestimate prevalence vs. single-item measures; licensing cost	Dated item wording; raw-vs-index cut-off confusion; less discriminating than newer multidimensional tools; best used as an initial screen
**Suitability for healthcare staff**	High—covers depression, anxiety and stress in one short administration	High for burnout specifically; pair with a distress measure for full coverage	Moderate–high as a quick depression screen; recommended alongside more comprehensive instruments

## Data Availability

No new data were created or analyzed in this study.

## Supplementary Materials

The following supporting information can be downloaded at: https://www.mdpi.com/article/10.3390/healthcare14132006/s1. Table S1: Full search strategy by database; Table S2: Quality assessment of principal psychometric and validation studies by instrument.

## Abbreviations

The following abbreviations are used in this manuscript:

DASS	Depression Anxiety Stress Scales
MBI	Maslach Burnout Inventory

## References

[1] Nagle E., Griskevica I., Rajevska O., Ivanovs A., Mihailova S., Skruzkalne I. (2024). Factors Affecting Healthcare Workers Burnout and Their Conceptual Models: Scoping Review. BMC Psychol..

[2] Amiri S., Mahmood N., Mustafa H., Javaid S.F., Khan M.A.B. (2024). Occupational Risk Factors for Burnout Syndrome Among Healthcare Professionals: A Global Systematic Review and Meta-Analysis. Int. J. Environ. Res. Public Health.

[3] Nagarajan R., Ramachandran P., Dilipkumar R., Kaur P. (2024). Global Estimate of Burnout Among the Public Health Workforce: A Systematic Review and Meta-Analysis. Hum. Resour. Health.

[4] Quesada-Puga C., Izquierdo-Espin F.J., Membrive-Jiménez M.J., Aguayo-Estremera R., Cañadas-De La Fuente G.A., Romero-Béjar J.L., Gómez-Urquiza J.L. (2024). Job Satisfaction and Burnout Syndrome Among Intensive-Care Unit Nurses: A Systematic Review and Meta-Analysis. Intensive Crit. Care Nurs..

[5] Kroenke K., Spitzer R.L., Williams J.B.W. (2001). The PHQ-9: Validity of a Brief Depression Severity Measure. J. Gen. Intern. Med..

[6] Spitzer R.L., Kroenke K., Williams J.B.W., Löwe B. (2006). A Brief Measure for Assessing Generalized Anxiety Disorder: The GAD-7. Arch. Intern. Med..

[7] Cohen S., Kamarck T., Mermelstein R. (1983). A Global Measure of Perceived Stress. J. Health Soc. Behav..

[8] Kristensen T.S., Borritz M., Villadsen E., Christensen K.B. (2005). The Copenhagen Burnout Inventory: A New Tool for the Assessment of Burnout. Work Stress.

[9] Jesus A., Pitacho L., Moreira A. (2023). Burnout and Suicidal Behaviours in Health Professionals in Portugal: The Moderating Effect of Self-Esteem. Int. J. Environ. Res. Public Health.

[10] Soares J.P., Lopes R.H., Mendonça P.B.S., Silva C.R.D.V., Rodrigues C.C.F.M., Castro J.L. (2023). Use of the Maslach Burnout Inventory Among Public Health Care Professionals: Scoping Review. JMIR Ment. Health.

[11] Lovibond S.H., Lovibond P.F. (1995). Manual for the Depression Anxiety Stress Scales.

[12] Moret-Tatay A., Pérez-Bermejo M., Murillo Llorente M.T., Moret-Tatay C., Esteve-Rodrigo J.V., De Stasio S. (2025). Comparing Short Versions of the Depression, Anxiety and Stress Scale (DASS): A Psychometric Study in the Italian General Population. Acta Psychol..

[13] Wang X., Cao C.H., Liao X.L., Jiang X.Y., Griffiths M.D., Chen I.H., Lin C.Y., Malas O. (2025). Comparing the Psychometric Evidence of the Depression, Anxiety, and Stress Scale-21 (DASS-21) between Spanish and Chinese Primary Schoolteachers: Insights from Classical Test Theory and Rasch Analysis. BMC Psychol..

[14] Fox R.S., Lillis T.A., Gerhart J., Hoerger M., Duberstein P. (2018). Multiple Group Confirmatory Factor Analysis of the DASS-21 Depression and Anxiety Scales: How Do They Perform in a Cancer Sample?. Psychol. Rep..

[15] Ali A.M., Alkhamees A.A., Hori H., Kim Y., Kunugi H. (2021). The Depression Anxiety Stress Scale 21: Development and Validation of the Depression Anxiety Stress Scale 8-Item in Psychiatric Patients and the General Public for Easier Mental Health Measurement in a Post COVID-19 World. Int. J. Environ. Res. Public Health.

[16] Makara-Studzińska M., Tyburski E., Załuski M., Adamczyk K., Mesterhazy J., Mesterhazy A. (2022). Confirmatory Factor Analysis of Three Versions of the Depression Anxiety Stress Scale (DASS-42, DASS-21, and DASS-12) in Polish Adults. Front. Psychiatry.

[17] Laranjeira C., Querido A., Sousa P., Dixe M.A. (2023). Assessment and Psychometric Properties of the 21-Item Depression Anxiety Stress Scale (DASS-21) among Portuguese Higher Education Students during the COVID-19 Pandemic. Eur. J. Investig. Health Psychol. Educ..

[18] Manzar M.D., Salahuddin M., Nureye D., Kashoo F.Z., Noohu M.M., Alotaibi J.S., Alamri M.S., Griffiths M.D. (2025). Depression, Anxiety, and Stress Scale-21 (DASS-21): Further Psychometric Exploration Using Robust Item Response Theory and Classical Theory Measures Among University Students. PLoS ONE.

[19] Yılmaz Koğar E., Koğar H. (2022). Using a Bifactor Exploratory Structural Equation Modeling Framework to Examine the Factor Structure of the Depression Anxiety and Stress Scales-21. Curr. Psychol..

[20] Kakemam E., Navvabi E., Albelbeisi A.H., Saeedikia F., Rouhi A., Majidi S. (2022). Psychometric Properties of the Persian Version of Depression Anxiety Stress Scale-21 Items (DASS-21) in a Sample of Health Professionals: A Cross-Sectional Study. BMC Health Serv. Res..

[21] Hilvano-Cabungcal A.M., Bonito S.R. (2025). Job-Related Factors Associated with Depression, Anxiety, and Stress among Healthcare Workers in a Tertiary Government Hospital in Metro Manila during the COVID-19 Pandemic. Acta Med. Philipp..

[22] Pachi A., Sikaras C., Melas D., Alikanioti S., Soultanis N., Ivanidou M., Ilias I., Tselebis A. (2025). Stress, Anxiety and Depressive Symptoms, Burnout and Insomnia Among Greek Nurses One Year After the End of the Pandemic: A Moderated Chain Mediation Model. J. Clin. Med..

[23] Maslach C., Jackson S.E. (1981). Maslach Burnout Inventory Manual.

[24] Lin C.-Y., Alimoradi Z., Griffiths M.D., Pakpour A.H. (2022). Psychometric Properties of the Maslach Burnout Inventory for Medical Personnel (MBI-HSS-MP). Heliyon.

[25] Bianchi R., Swingler G., Schonfeld I.S. (2024). The Maslach Burnout Inventory Is Not a Measure of Burnout. Work.

[26] Knox M., Willard-Grace R., Huang B., Grumbach K. (2018). Maslach Burnout Inventory and a Self-Defined, Single-Item Burnout Measure Produce Different Clinician and Staff Burnout Estimates. J. Gen. Intern. Med..

[27] Zung W.W.K. (1965). A Self-Rating Depression Scale. Arch. Gen. Psychiatry.

[28] Jokelainen J., Timonen M., Keinänen-Kiukaanniemi S., Härkönen P., Jurvelin H., Suija K. (2019). Validation of the Zung Self-Rating Depression Scale (SDS) in Older Adults. Scand. J. Prim. Health Care.

[29] Lu G., Xiao S., He J., Xie W., Ge W., Meng F., Yang Y., Yu S., Liu R. (2023). Prevalence of Depression and Its Correlation with Anxiety, Headache and Sleep Disorders among Medical Staff in the Hainan Province of China. Front. Public Health.

[30] Li G., Wei C., Fang K., Jiang H., Liu Q., Ou J. (2024). The Correlated Factors of Anxiety and Depression among Chinese Hospital Staff during the COVID-19 Local Outbreak. Medicine.

[31] Zhang P., Tang L., Ciren D. (2023). Anxiety and Depression Survey and Analysis of Hospital Staff in a Designated Hospital in Shannan City During the COVID-19 Pandemic. Med. Sci. Monit. Basic Res..

[32] Li S., Shang S., Wang J., Yang B., Jiang W. (2023). Research on the Psychological Status of Medical Staff during the COVID-19 Epidemic in China: A Longitudinal Study. Medicine.

[33] Dunstan D.A., Scott N. (2019). Clarification of the Cut-Off Score for Zung’s Self-Rating Depression Scale. BMC Psychiatry.

[34] Cheng L., Gao W., Xu Y., Yu Z., Wang W., Zhou J., Zang Y. (2023). Anxiety and Depression in Rheumatoid Arthritis Patients: Prevalence, Risk Factors and Consistency between the Hospital Anxiety and Depression Scale and Zung’s Self-Rating Anxiety Scale/Depression Scale. Rheumatol. Adv. Pract..

[35] Dunstan D.A., Scott N., Todd A.K. (2017). Screening for Anxiety and Depression: Reassessing the Utility of the Zung Scales. BMC Psychiatry.

