# Supplementary material for: Assessment of Mental Health in Healthcare Personnel: A Review of DASS, MBI, and Zung Scales
Source: Healthcare (Basel). 2026 Jul 6;14(13):2006. doi: 10.3390/healthcare14132006 (PMC13361314; doi:10.3390/healthcare14132006)
Supplement: Supplementary file 1 [file healthcare-14-02006-s001.zip › healthcare-4351470-supplementary.pdf]

Supplementary Materials

Table S1. Full electronic search strategy for each database (final search of all databases: 26 June 2026). Search terms were combined with the Boolean operators AND and OR; an asterisk (*) denotes truncation, [tiab] a title/abstract field tag, [MeSH] a Medical Subject Heading, TITLE-ABS-KEY a Scopus field code, and TS a Web of Science topic field.

#	Database	Full search string	Date
1	PubMed / MEDLINE	(("DASS"[tiab] OR "Depression Anxiety Stress Scale*"[tiab]) OR ("Maslach Burnout Inventory"[tiab] OR "MBI"[tiab]) OR ("Zung Self-Rating Depression Scale"[tiab] OR "Zung SDS"[tiab] OR "Self-Rating Depression Scale"[tiab] OR "SDS"[tiab])) AND ("Health Personnel"[MeSH] OR "health* worker*"[tiab] OR "health* personnel"[tiab] OR "nurse*"[tiab] OR "physician*"[tiab] OR "medical staff"[tiab] OR "hospital staff"[tiab] OR "hospital employee*"[tiab]) AND ("depression"[MeSH] OR "anxiety"[MeSH] OR "stress, psychological"[MeSH] OR "burnout, professional"[MeSH])	26 June 2026
2	Scopus	TITLE-ABS-KEY(("DASS" OR "Depression Anxiety Stress Scale*") OR ("Maslach Burnout Inventory" OR "MBI") OR ("Zung Self-Rating Depression Scale" OR "Zung SDS" OR "Self-Rating Depression Scale" OR "SDS")) AND TITLE-ABS-KEY("health* worker*" OR "health* professional*" OR "nurse*" OR "physician*" OR "medical staff" OR "hospital staff" OR "hospital employee*") AND TITLE-ABS-KEY("depression" OR "anxiety" OR "stress" OR "burnout*")	26 June 2026
3	Web of Science (Core Collection)	TS=(("DASS" OR "Depression Anxiety Stress Scale*") OR ("Maslach Burnout Inventory" OR "MBI") OR ("Zung Self-Rating Depression Scale" OR "Zung SDS" OR "Self-Rating Depression Scale" OR "SDS")) AND TS=("health* worker*" OR "health* professional*" OR "nurse*" OR "physician*" OR "medical staff" OR "hospital staff" OR "hospital employee*") AND TS=("depression" OR "anxiety" OR "stress" OR "burnout*")	26 June 2026
4	ScienceDirect	("DASS" OR "Depression Anxiety Stress Scales") AND ("Maslach Burnout Inventory" OR "MBI") AND ("Zung Self-Rating Depression Scale" OR "Self-Rating Depression Scale") AND ("healthcare workers" OR "nurses" OR "physicians" OR "hospital staff") AND ("depression" OR "anxiety" OR "stress" OR "burnout")	26 June 2026
5	Google Scholar	("DASS" OR "Maslach Burnout Inventory" OR "Zung Self-Rating Depression Scale" OR "Self-Rating Depression Scale") AND ("healthcare workers" OR "nurses" OR "physicians" OR "hospital staff") AND ("depression" OR "anxiety" OR "stress" OR "burnout")	26 June 2026

Table S2. Quality assessment of principal psychometric and validation studies organised by instrument.

#	Study (author, year)	Instrument / version	Population & setting	n	Design	Reliability (Cronbach α)	Validity evidence / key finding	Relevance to HCW	Overall quality
**Depression, Anxiety and Stress Scales (DASS)**									
1	Moret-Tatay et al., 2025 **[12]**	DASS-21/-12/-10/-8	General population, Italy	NR	Cross-sectional psychometric comparison	High across all short forms	DASS-8 best model fit and explained variance; DASS-12 best brevity–clarity compromise	Low	Moderate
2	Wang et al., 2025 **[13]**	DASS-21	Primary-school teachers, Spain & China	NR	Cross-cultural psychometric (CTT & Rasch)	Acceptable in both countries	Factor structure culture-dependent (one-factor China, three-factor Spain)	Low	Moderate
3	Fox et al., 2018 **[14]**	DASS-21	Cancer patients and non-cancer controls, USA	376 + 207	Multiple-group CFA	Satisfactory internal reliability	Stable three-factor structure in both groups; convergent validity (suicidality, QoL, self-rated health)	Low	High
4	Ali et al., 2021	DASS-8 (from DASS-21)	Psychiatric patients & general public, Saudi Arabia	Large (NR)	Scale development & validation	High internal consistency	DASS-8 most stable short form; distinguishes clinical from non-clinical as well as full scale	Moderate	High
5	Makara-Studzińska et al., 2022 **[16]**	DASS-42/-21/-12	General adults, Poland	NR	Confirmatory factor analysis	High internal consistency	Good three-factor structure across all three versions	Low	High
6	Laranjeira et al., 2023 **[17]**	DASS-21	Higher-education students, Portugal	NR	Cross-sectional validation	High internal consistency	Good validity; depression, anxiety and stress clearly differentiated	Low	Moderate
7	Manzar et al., 2025 **[18]**	DASS-21	University students	NR	Psychometric (IRT & CTT)	Good internal consistency	High factorial validity; strong discrimination across severity levels	Low	High
8	Yılmaz Koğar & Koğar, 2023 **[19]**	DASS-21	Adult sample	NR	Bifactor exploratory SEM	Good internal consistency	Robust structure dominated by a general distress factor	Low	High
9	**Kakemam et al., 2022 [20]**	DASS-21 (Persian)	Hospital nurses, Iran (public hospitals)	1,135	CFA validation + test–retest	α = 0.93 (D), 0.91 (S), 0.79 (A); ICC 0.75–0.86	CFA confirmed three-factor structure (RMSEA 0.078, CFI 0.917, TLI 0.906)	High	High

#	Study (author, year)	Instrument / version	Population & setting	n	Design	Reliability (Cronbach α)	Validity evidence / key finding	Relevance to HCW	Overall quality
10	**Pachi et al., 2025 [22]**	DASS-21 (+ CBI, AIS)	Hospital nurses, Greece (post-pandemic)	380	Cross-sectional; mediation model	Applied use (NR)	Applied screening: depression 35%, anxiety 33.3%, stress 33.9%; DASS-21 used with CBI	High	Moderate
11	**Hilvano-Cabungcal & Bonito, 2025 [21]**	DASS-21	HCWs (doctors, nurses, allied), tertiary hospital, Philippines	364	Analytical cross-sectional	Applied use (NR)	Applied screening: depression 49.2%, anxiety 61.5%, stress 30.2%	High	Moderate
**Maslach Burnout Inventory (MBI)**									
12	Soares et al., 2023 **[10]**	MBI (HSS)	Public health-care professionals (scoping review)	NR	Scoping review	—	Synthesised MBI use among public-health workers; supports early detection	High	Moderate
13	Lin et al., 2022 **[24]**	MBI-HSS-MP	Medical personnel; Taiwan, Iran, UK & Sweden	NR	Multi-country psychometric validation	Reliable across sites	Confirmed three-factor structure (EE, DP, PA); universal across genders and roles	High	High
14	Bianchi et al., 2024 **[25]**	MBI (critique)	Conceptual / critical analysis	—	Critical / conceptual review	—	Argues the MBI over-emphasises emotional exhaustion; no new psychometric data	High	Limited
15	Knox et al., 2018 **[26]**	MBI vs single-item measure	Physicians & clinical/administrative staff, UCSF, USA	NR	Cross-sectional comparative	—	MBI underestimated burnout relative to a self-defined single-item measure	High	Moderate
**Zung Self-Rating Depression Scale (SDS)**									
16	Jokelainen et al., 2019 **[28]**	Zung SDS	Older adults, Finland & Estonia	NR	Validation study (vs BDI)	Good internal consistency	Good validity against the BDI; effective primary-care screening tool	Moderate	High
17	Dunstan & Scott, 2019 **[33]**	Zung SDS	Methodological (cut-off) study	NR	Cut-off determination	—	Cut-off ≥40 distinguishes depressed from non-depressed; sensitivity/specificity discussed	Moderate	Moderate

#	Study (author, year)	Instrument / version	Population & setting	n	Design	Reliability (Cronbach α)	Validity evidence / key finding	Relevance to HCW	Overall quality
18	Cheng et al., 2023 **[34]**	Zung SAS/SDS vs HADS	Rheumatoid arthritis patients, China	NR	Cross-sectional comparative	—	Good consistency between HADS and Zung SDS	**Low**	**Moderate**
19	Dunstan et al., 2017 **[35]**	Zung SAS/SDS	Methodological reassessment (screening utility)	NR	Methodological review	—	Effective for screening but require updating; useful as an initial step	**Moderate**	**Moderate**
20	**Lu et al., 2023 [29]**	Zung SDS (+ SAS, PSQI)	Medical staff, 4 hospitals, China (Hainan)	645	Cross-sectional; stratified cluster sampling	SDS $\alpha \approx 0.81$	Applied screening: 1-year depression prevalence 42.7% (higher in nurses, women, shift workers)	**High**	**Moderate**
21	**Li G. et al., 2024 [30]**	Zung SDS (+ SAS)	Hospital staff, China (local COVID-19 outbreak)	800	Cross-sectional	Applied use (NR)	Applied screening: depression 35.1% (nurses > doctors)	**High**	**Moderate**
22	**Zhang et al., 2023 [31]**	Zung SDS (+ SAS)	Hospital staff, designated COVID-19 hospital, China (Tibet)	267	Cross-sectional	Applied use (NR)	Applied screening: depression 63.67%, anxiety 36.70%	**High**	**Moderate**
23	**Li S. et al., 2023 [32]**	Zung SDS (+ SAS)	Medical staff, Wuhan, China	NR	Longitudinal	Applied use (NR)	Applied screening: longitudinal tracking of anxiety and depression over time	**High**	**Moderate**
